# Mortality related to hospital-associated infections in a tertiary hospital; repeated cross-sectional studies between 2004-2011

**DOI:** 10.1186/s13756-015-0097-9

**Published:** 2015-12-29

**Authors:** Anne Mette Koch, Roy Miodini Nilsen, Hanne Merete Eriksen, Rebecca Jane Cox, Stig Harthug

**Affiliations:** Department of Research and Development, Haukeland University Hospital, Jonas Liesv. 65, 5021 Bergen, Norway; Department of Clinical Science, University of Bergen, Jonas Liesv. 87, Bergen, Norway; Norwegian Institute of Public Health, Postboks 4404, Nydalen, 0403 Oslo, Norway; K.G Jebsen Centre for Influenza Vaccine Research, Department of Clinical Science, University of Bergen, Jonas Lies v. 87, Bergen, Norway

**Keywords:** Hospital associated infections, HAIs, Mortality, Prevalence, Blood stream infection, Lower respiratory tract infection

## Abstract

**Background:**

Hospital-associated infections (HAIs) are reported to increase patient mortality and incur longer hospital stays. Most studies to date have focused on specific groups of hospitalised patients with a rather short follow-up period. In this repeated cross-sectional study, with prospective follow-up of 19,468 hospitalized patients, we aimed to analyze the impact of HAIs on mortality 30 days and 1 year after the prevalence survey date.

**Methods:**

The study was conducted at Haukeland University Hospital, Norway, a large combined emergency and referral teaching hospital, from 2004 to 2011 with follow-up until November 2012. Prevalence of all types of HAIs including urinary tract infections (UTI), lower respiratory tract infections (LRTI), surgical site infections (SSI) and blood stream infections (BSI) were recorded four times every year. Information on the date of birth, admission and discharge from the hospital, number of diagnoses (ICD-10 codes) and patient’s mortality was retrieved from the patient administrative data system.

The data were analysed by Kaplan-Meier survival analysis and by multiple Cox regression analysis, adjusted for year of registration, time period, sex, type of admission, Charlson comorbidity index, surgical operation, use of urinary tract catheter and time from admission to the prevalence survey date.

**Results:**

The overall prevalence of HAIs was 8.5 % (95 % CI: 8.1, 8.9). Patients with HAIs had an adjusted hazard ratio (HR) of 1.5 (95 % CI: 1.3, 1.8,) and 1.4 (95 % CI: 1.2, 1.5) for death within 30-days and 1 year, relative to those without HAIs. Subgroup analyses revealed that patients with BSI, LRTI or more than one simultaneous infection had an increased risk of death.

**Conclusions:**

In this long time follow-up study, we found that HAIs have severe consequences for the patients. BSI, LRTI and more than one simultaneous infection were independently and strongly associated with increased mortality 30 days and 1 year after inclusion in the study.

## Background

In industrialized countries, at any given time, more than one out of twenty patients has a hospital associated infection (HAI) [1–7]. Even if great efforts have been made to reduce HAIs during the last decades, such infections are still among the most common complications affecting hospitalized patients [8]. The risk for HAIs depends on patient related factors, various invasive procedures and treatment provided during hospital stay. Medical technology and treatment are becoming more complex every year and more patients with severe underlying diseases are treated. Consequently, HAIs vary according to the type of clinical department, with the highest infection rate usually found in intensive care units (ICU), neonatal and burn units [3, 5, 6, 9, 10].

HAIs affect a large number of patients in terms of complications, increased mortality and longer hospital stay. HAIs may also affect the quality of life like long term disability and psychological trauma and are reported as one of the top leading causes of in-hospital deaths worldwide [8, 11]. HAIs also impose significant economic consequences on the healthcare system [12–14].

The association between different HAIs and mortality is well established in previously published studies [14–19], and such associations are particularly found in patients with lower respiratory tract (LRTI) [14, 16, 17] and blood stream infections (BSI) [14, 16, 18, 20]. However, some of the studies are primarily performed in high risk units, with a small number of patients, focusing on one type of HAI, or without taking co-morbidity into account.

In this study the purpose was to evaluate a possible relationship between various types of HAIs and the risk of mortality within 30 days and 1 year among 19,468 patients in a combined emergency and referral hospital in Norway.

## Methods

### Setting

The study was conducted at Haukeland University Hospital, a hospital trust including a large somatic hospital and psychiatric hospital, a smaller emergency hospital, and a specialized orthopedic hospital. All together the hospital has approximately 1000 somatic beds. It covers about one million inhabitants in Western Norway, and is also an emergency hospital for 300,000 people. It provides all specialties apart from organ transplants, and it includes large intensive care units with approximately 30 beds, a neonatal unit with 7 beds, and a national burns center with 5 beds.

### Method

**Fig. 1:**
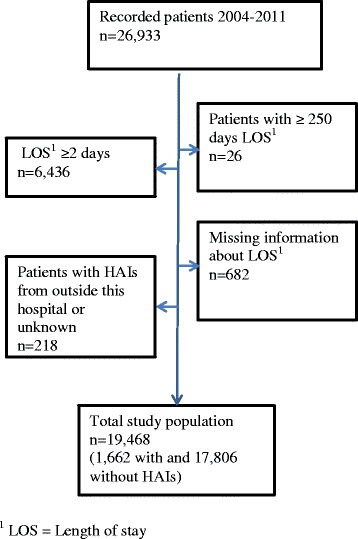
Flow-chart showing patient inclusion in the study

The study was designed as a repeated cross-sectional study with prospective follow-up of life status. Data collection was performed four times annually from November 2004 to November 2011, with a one year follow up for all subjects up to November 2012. All in-patients on the day of prevalence survey were included in the study and a total of 26,933 patients were recorded following 32 different surveys. When excluding patients with hospital stay less than 2 days (by definition not at risk for HAI) or longer than 250 days, as well as patients with HAIs transferred from other hospitals and patients with missing information on LOS, we ended up with a patient cohort of 19,468 (Fig. 1). When a patient had more than one registration in the surveillance system during the follow-up period, only the first admission was included.

### Ethics

The data was collected as a part of the hospital’ s infection prevalence survey. According to the Health Research Act, Norway, quality assurance projects, surveys and evaluations that are intended to ensure that diagnosis and treatment actually produce the intended results do not need ethical committee approval and patient consent is not required. Hence, the study was only approved by the hospital’ s privacy ombudsman [Ref: 2013/9818].

### Data collection

The Department of Infection Control has the overall responsibility for data collection in a local registry established for the mandatory infection surveillance, which was linked to the patient administrative system. On the day of prevalence survey, dedicated nurses or physicians in the somatic wards reviewed all in-patients in an on-line system. The patients’ infection status was identified, and the inclusion day was defined as the day of the prevalence survey. HAIs were identified according to a simplified version of the definitions developed and recommended by the Centres for Disease Control and prevention (CDC), USA [2, 21].

HAIs were defined as any infection identified at least 48 h after hospital admission without evidence of the infection being present or incubating at the time of admission. All in-patients registered in somatic wards at the hospital at 8 a.m. on the designated day were included in the survey.

All types of HAIs were recorded and analysed, including symptomatic urinary tract infection (UTI), lower respiratory tract infection (LRTI), blood stream infection (BSI), and surgical site infection (SSI). HAIs with only a few included cases such as skin, soft-tissue infections and gastrointestinal infections were analysed together as “other infections”. Patients with more than one type of infection simultaneously were analysed as a separate group. The prevalence of surgical site infections was analysed including all patients for overall prevalence and among operated patients only in the remaining analysis.

The following variables were recorded for each patient: sex, age, season of admission (spring, summer, autumn and winter), elective versus emergency admission, surgical procedure, use of urinary tract catheter (permanent and intermittent catheter) and antibiotic therapy. Date of admission was automatically collected from the patient administrative data system.

Up to seven diagnoses according to ICD-10 (The international classification of Diseases, ICD-10) were recorded for each patient at discharge. All diagnoses were weighted according to Charlson comorbidity index, a method validated to predict mortality by classifying or weighting the patient’s comorbid conditions [22, 23]. Information about mortality was recorded from patient administrative data system 30 days and 1 year after patient’s inclusion in the study (the day of the prevalence survey).

### Statistical analysis

HAIs were analysed as a binary exposure variable (no HAI, any HAI). We also analysed HAIs by type of infection (no HAI, UTI, LRTI, BSI, other HAIs, multiple HAIs, SSI), which were mutually exclusive.

Descriptive statistics were used to quantify sample characteristic whereas the Kaplan Meier survivor function were used to describe the percentage of survivors after 30 days and 1 year after infection status. To test for difference in survival functions across HAI categories, we used the log-rank test. We further estimated the associations of HAIs with 30 days and 1 year mortality as hazard ratios with 95 % confidence intervals (CIs) using Cox regression models. The time from study inclusion (i.e., date of prevalence survey) until death was used as the measure of event free time. All patients were monitored for up to 30 and 1 year. The hazard ratios were estimated by crude models as well as after controlling for year of inclusion (continuous), time period (categorical calendar quarters), patient’s sex (woman, man), patient’s age (continuous), type of admission to hospital (acute, elective), surgical operation (no, yes), and use of urinary tract catheter (no, intermittent/permanent). We additionally adjusted for time from hospital admission to study inclusion (i.e., the pre-prevalence period) and Charlson comorbidity index. Because these two variables formed a non-linear relationship with mortality or infection status, they were categorized and included as categorical model terms (6 categories each) to achieve better adjustment. All covariates were chosen because they have previously been strongly related with mortality of HAIs. Finally, by visual inspection of the log-log plot of survival, we verified that the proportional-hazards assumption was essentially fulfilled for all variables in the models. All analyses were performed using Stata/IC version 14.0 (StataCorp, Texas, USA) for Windows. All *P* values were two sided and values below 0.05 were considered statistically significant.

## Results

### Patient characteristics and prevalence of HAIs

During the study period 19,468 patients were included, 1662 patients had HAIs and the remaining 17,806 did not have HAIs. The overall prevalence of HAIs was 8.5 % and the prevalence of the four most frequently recorded types of infections was for LRTI 2.2 %, UTI 2.1 %, BSI 0.5 %, and SSI 1.6 %. Prevalence among operated patients was 4.5 % (Table 1). A general overview of the analysed variables is shown in Table 2. Fifty-three percent of the patients were females. The overall prevalence was higher in males than in females (9.7 % vs. 7.5 %) and increased with age. For the oldest patients (>74 years old), we found a prevalence of 11.3 % vs. 2.6 % for the youngest patients (<14 years). A total of 6925 (35.6 %) patients had undergone surgery and the prevalence of HAIs among operated patients was 15.0 % compared to 5 % for the non-operated patients. Acute admission patients had a higher prevalence of HAIs than those with elective admission, 9.6 % and 6.8 %, respectively. Seventeen percent of the patients had urinary tract catheters (13.8 % permanent and 2.9 % intermittent) and 26.2 % of the patients received antibiotics. We found an association between hospital stay before the date of prevalence study and the prevalence of HAIs. Charlson comorbidity index up to 3 was associated with a higher prevalence of HAI, whereas patients with a Charlson index 4 or higher had a lower prevalence (Table 2).

**Table 1:** Prevalence of HAIs among 19,468 patients at Haukeland University hospital, 2004-2011

Type of infection	n	% (95 % CI)
All infections	1662	8.5 % (95 % CI: 8.1, 8.9)
Urinary tract	407	2.1 % (95 % CI 1.9, 2.3)
Lower respiratory	428	2.2 % (95 % CI: 2.0, 2.4)
Blood stream	89	0.5 % (95 CI: 0.4, 0.6)
Surgical site	311	1.6 % (95 % CI: 1.4, 1.8)
Surgical site^a^	311	4.5 % (95 % CI: 4.2, 4.8)

^a^Among 6925 operated patients

**Table 2:** Characteristics of 19,468 patients with and without hospital-associated infections (HAIs) treated at Haukeland University Hospital, 2004-2011

		HAIs			Prevalence of HAIs
	All patients	No	Yes		
Characteristics	n (%)	n (%)	n (%)	*P* value^a^	%
All	19468 (100)	17806 (100.0)	1662 (100.0)		8.5
Gender				<0.001	
Women	10140 (52.1)	9378 (52.7)	762 (45.8)		7.5
Men	9328 (47.9)	8428 (47.3)	900 (54.2)		9.7
Age (years)				<0.001	
0-14	2131 (10.9)	2076 (11.7)	55 (3.3)		2.6
15-34	2447 (12.6)	2331 (13.1)	116 (7.0)		4.7
35-54	3345 (17.2)	3084 (17.3)	261 (15.7)		7.8
55-74	6113 (31.4)	5498 (30.9)	615 (37.0)		10.1
>74	5432 (27.9)	4817 (27.1)	615 (37.0)		11.3
Time period				0.005	
Jan-Mar	4924 (25.3)	4512 (25.3)	412 (24.8)		8.4
Apr-Jun	5010 (25.7)	4618 (25.9)	392 (23.6)		7.8
Jul-Sept	4786 (24.6)	4391 (24.7)	395 (23.8)		8.3
Oct-Dec	4748 (24.4)	4285 (24.1)	463 (27.9)		9.8
Admission type				<0.001	
Acute	12080 (62.3)	10918 (61.6)	1162 (70.2)		9.6
Elective	7304 (37.7)	6810 (38.4)	494 (29.8)		6.8
Surgery				<0.001	
No	12543 (64.4)	11920 (66.9)	623 (37.5)		5.0
Yes	6925 (35.6)	5886 (33.1)	1039 (62.5)		15.0
Urinary tract					
catheter				<0.001	
No	16216 (83.3)	15162 (85.2)	1054 (63.4)		6.5
Yes, permanent	2682 (13.8)	2150 (12.1)	532 (32.0)		19.8
Yes, intermittent	570 (2.9)	494 (2.8)	76 (4.6)		13.3
Use of antibiotics				<0.001	
No	14372 (73.8)	14241 (80.0)	131 (7.9)		0.9
Yes	5096 (26.2)	3565 (20.0)	1531 (92.1)		30.0
Pre-prevalence period (days)^b^				<0.001	
2	5182 (26.6)	5112 (28.7)	70 (4.2)		1.4
3-5	4537 (23.3)	4339 (24.4)	198 (11.9)		4.4
6-9	4133 (21.2)	3756 (21.1)	377 (22.7)		9.1
10-15	2295 (11.8)	1938 (10.9)	357 (21.5)		15.6
16-30	2258 (11.6)	1814 (10.2)	444 (26.7)		19.7
>30	1063 (5.5)	847 (4.8)	216 (13.0)		20.3
Charlson comorbidity index^c^				<0.001	
0	9758 (50.1)	9202 (51.7)	556 (33.5)		5.7
1	3464 (17.8)	3125 (17.6)	339 (20.4)		9.8
2	3234 (16.6)	2827 (15.9)	407 (24.5)		12.6
3	1118 (5.7)	951 (5.3)	167 (10.0)		14.9
4	420 (2.2)	377 (2.1)	43 (2.6)		10.2
>4	1380 (7.1)	1236 (6.9)	144 (8.7)		10.4

^a^By chi-square test

^b^Time from hospital admission to study inclusion

^c^Information was missing for 94 patients on Charlson comorbidity index and 84 on admission type

### Thirty day and 1 year mortality

Table 3 shows 30 day and 1 year mortality for all patients according to patient characteristics. Of all patients 909 (4.7 %) died within 30 days and 3188 (16.4 %) within 1 year. We found that mortality was higher among men than women, whereas mortality increased with age for both men and women. Patients with acute admission to the hospital had higher mortality than patients with elective admission. Increased mortality was also related to a longer pre-prevalence period, with an exception for patients having a pre-prevalence stay of more than 30 days. A high Charlson comorbidity index also gave increased mortality, and for patients with a Charlson index > 4 we found that 17.0 % and 61.4 % died within 30 days and 1 year, respectively.

**Table 3:** Thirty day and 1 year mortality according to characteristics of 19,468 patients treated at Haukeland University Hospital, 2004-2011

	30-days mortality,	1 year mortality,
Characteristics	n (%)	n (%)
All	909 (4.7)	3188 (16.4)
Gender		
Women	413 (4.1)	1449 (14.3)
Men	496 (5.3)	1739 (18.6)
Age (years)		
0-14	7 (0.3)	19 (0.9)
15-34	8 (0.3)	45 (1.8)
35-54	46 (1.4)	259 (7.7)
55-74	309 (5.1)	1172 (19.2)
>74	539 (9.9)	1693 (31.2)
Time period		
Jan-Mar	221 (4.5)	783 (15.9)
Apr-Jun	225 (4.5)	822 (16.4)
Jul-Sept	232 (4.9)	794 (16.6)
Oct-Dec	231 (4.9)	789 (16.6)
Admission type		
Acute	801 (6.6)	2477 (20.5)
Elective	108 (1.5)	705 (9.7)
Surgery		
No	739 (5.9)	2526 (20.1)
Yes	170 (2.5)	662 (9.6)
Urinary tract		
catheter		
No	526 (3.2)	2288 (14.1)
Yes, permanent	360 (13.4)	818 (30.5)
Yes, intermittent	23 (4.0)	82 (14.4)
Use of antibiotics		
No	512 (3.6)	2035 (14.2)
Yes	397 (7.8)	1153 (22.6)
Pre-prevalence		
period (days)^a^		
2	100 (1.9)	505 (9.8)
3-5	180 (4.0)	558 (12.3)
6-9	209 (5.1)	750 (18.2)
10-15	163 (7.1)	555 (24.2)
16-30	182 (8.1)	583 (25.8)
>30	75 (7.1)	237 (22.3)
Charlson comorbidity index^b^		
0	93 (1.0)	331 (3.4)
1	150 (4.3)	462 (13.3)
2	249 (7.7)	925 (28.6)
3	134 (12.0)	449 (40.2)
4	49 (11.7)	168 (40.0)
>4	234 (17.0)	847 (61.4)

^a^Time from hospital admission to study inclusion

^b^Information was missing for 94 patients on Charlson comorbidity index and 84 on admission type

Among patients with HAIs 10.8 % (95 % CI: 9.3, 12.3) died within the first month after they were included in the study compared to 4.1 % (95 % CI: 3.8, 4.4) in patients without HAIs. Within 1 year 28.4 % (95 % CI: 26.2, 30.6) with HAIs and 15.3 % (95 % CI: 14.7, 15.8) without HAIs had died.

**Fig. 2:**
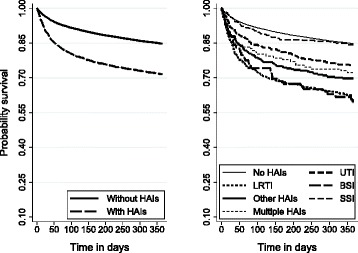
Survival of 19,468 patients with and without hospital-associated infections (HAIs)

By Kaplan-Meier survival analyses we found that patients without HAIs had a 1 year survival of 70 %, compared to 85 % in those without HAIs (*p* < 0.001). The lowest survival rates were found among patients with LRTI and BSI. Patients with SSI had the same survival rates as those without HAIs (Fig. 2).

Following adjustment for confounding factors we found that patients with HAIs had a significantly increased mortality risk compared to patients without HAIs. Within 30 days and 1 year, patients with HAIs had an adjusted hazard ratio (HR) of 1.5 (95 % CI: 1.3, 1.8) and 1.4 (95 % CI: 1.2, 1.5) for death, respectively, relative to those without HAIs. The highest mortality risk was observed in patients with BSI, followed by patients with LRTI. No increased risk of death was found in patients with UVI and SSI during the follow up periods (Table 4).

**Table 4:** Mortality in 19,468 patients with hospital-associated infections at Haukeland University Hospital, 2004-2011

		30-days mortality			1-year mortality		
	Infections	Mortality	Crude hazard ratio	Adjusted hazard ratio	Mortality	Crude hazard ratio	Adjusted hazard ratio
Type of infection	n	n (%)	(95 % CI)^a^	(95 % CI)^b^	n (%)	(95 % CI)^a^	(95 % CI)^b^
All infections	1662	180 (10.8)	2.7 (2.3, 3.2)	1.5 (1.3, 1.8)	472 (28.4)	2.1 (1.9, 2.3)	1.4 (1.2, 1.5)
Urinary tract	407	36 (8.9)	2.2 (1.6, 3.1)	0.9 (0.6, 1.3)	100 (24.6)	1.7 (1.4, 2.1)	0.9 (0.7, 1.1)
Lower respiratory	428	68 (15.9)	4.1 (3.2, 5.3)	1.9 (1.5, 2.5)	161 (37.6)	3.0 (2.5, 3.5)	1.7 (1.4, 2.0)
Blood stream	89	11 (12.4)	3.1 (1.7, 5.7)	2.7 (1.5, 4.9)	36 (40.5)	3.1 (2.3, 4.4)	3.0 (2.1, 4.1)
Other	301	36 (12.0)	3.1 (2.2, 4.3)	1.7 (1.2, 2.4)	91 (30.2)	2.2 (1.8, 2.8)	1.5 (1.2, 1.9)
>1 infection	126	16 (12.7)	3.3 (2.0, 5.4)	2.6 (1.5, 4.3)	35 (27.8)	2.0 (1.5, 2.8)	1.8 (1.3, 2.6)
Surgical site^c^	311	13 (4.2)	2.4 (1.3, 4.2)	1.3 (0.7, 2.3)	49 (15.8)	2.1 (1.6, 2.8)	1.2 (0.9, 1.6)

^a^Estimated by Cox regression model

^b^Adjusted by year and calendar period of prevalence survey, patient’s sex and age, type of admission, surgery operation, use of urinary tract catheter, time from hospital admission to study inclusion (pre-prevalence period), and Charlson comorbidity index

^c^Among 6925 operated patients

## Discussion

The main findings in this study were that patients with HAIs had a higher risk of dying within 30 days and 1 year, compared to those without HAIs. BSI, LRTI and having more than one HAIs simultaneously were associated with increased mortality, whereas patients with SSI and UTI did not have an increased risk of dying. The prevalence of HAIs was higher than previously reported from our hospital [7]. The reason for this might be that patients by definition are not at risk of acquiring infection during the first two days in hospital and that all patients with less than two days length of stay were excluded from this study.

Only a few studies have estimated the global impact of HAIs on mortality in hospital, and as in this study, they are all reported increased mortality [14, 16, 17, 24]. Comparison of the results between studies remains difficult since different methods are used in the various studies. However, in a study by Kanerva et al., based on prevalence survey data from more than 7000 patients, 28 day mortality rate for patients with HAIs was slightly lower than the 30 day mortality found in our study, 9.8 % and 10.8 % respectively [17].

As shown in other studies, we also found that both patients with BSI and LRTI had increased risk of dying during the follow-up period [14, 16]. Patients with SSI had no increased mortality risk, the same result has also been shown in other studies [16, 17]. We could not confirm that UTIs led to increased mortality, which contrasts with the findings from Fabbro-Peray et al. who reported OR for death after 60 days to be 1.6 (95 % CI: 1.3-2.1) [16].

We identified several patient characteristics which increased the risk of HAIs and death. Male gender, old age, use of urinary tract catheter, longer pre-prevalence period, and comorbidity were all factors affecting patient outcome. These factors should always be taken into account in assessing each patient’s risk of HAIs, and in targeting infection control and prevention measures in care and treatment.

To adjust for comorbidity we used the Charlson comorbidity index [22, 23]. An alternative method for adjusting risk of death would have been McCabe score, which assess patients subjectively in three different groups (non-fatal, ultimately fatal and rapidly fatal illness). According to other studies there is a significant correlation between Charlson index and McCabe class, although McCabe classifications are assumed to have a better goodness-of-fit for predicting death [16, 17]. McCabe classifications were, however, not part of the data set in the prevalence surveys in our hospital, and for this reason the Charlson index, which was already available in the patient administrative system, was utilised.

Our study has some limitations. Many people have been involved in data collection, and in spite of written information and validated definitions, different practices and assessments may have influenced the results. Furthermore, we did not investigate if patients without an infection on the day of surveillance had a HAI later on during the hospital stay. This might have resulted in misclassification and an underestimation of the impact of HAIs on mortality.

A possible sample bias may also have occurred since 457 out of 26,833 patents were excluded due to implausible data (Fig. 1). However, since the number of excluded patients was relatively small, we do not assume that this lead to a systematic bias.

We have no information about the length of stay from admission to onset of HAI, and have used the time from admission to prevalence survey (the pre-prevalence period) as a surrogate for this. Especially for types of infection with long duration, such as SSI, the infection may have started several days before the prevalence survey. Follow-up time will therefore be longer than 30 days and 1 year, and possibly different for the various types of HAIs.

Even if Charlson index is described as an appropriate tool to adjust for comorbidity, the use of ICD-codes has some limitations. The sensitivity of ICD codes has varied in published studies according to different practice of coding in different hospitals and countries [25, 26]. During the long study period it is also possible that some changes in practices for coding have occurred in our hospital, although we have not identified any extensive changes in these practices.

## Conclusion

In this longitudinal study based on prevalence data from a large emergency and referral teaching hospital in Norway, we found that HAIs have severe consequences for patients. BSI, LRTI or more than one simultaneous HAI were independently and strongly associated with increased mortality 30 days and 1 year after inclusion in the study. Routinely collected prevalence surveillance data, integrated with patient administrative system, is of great value as a basis for studying long term consequences of HAIs.
